# Extramedullary Plasmacytoma Diagnosed in an HIV-Positive Patient by an Unusual Clinical Presentation

**DOI:** 10.1155/2016/6305173

**Published:** 2016-11-17

**Authors:** Paulo de Camargo Moraes, Luiz Alexandre Thomaz, Victor Angelo Martins Montalli, José Luiz Cintra Junqueira, Camila Maria Beder Ribeiro, Luciana Butini Oliveira

**Affiliations:** ^1^Department of Oral Surgery, School of Dentistry, SLMandic, Campinas, SP, Brazil; ^2^Department of Oral Radiology, School of Dentistry, SLMandic, Campinas, SP, Brazil; ^3^Department of Oral Pathology, School of Dentistry, SLMandic, Campinas, SP, Brazil; ^4^Department of Oral Pathology and Stomatology, FOP-UNICAMP, Piracicaba, SP, Brazil

## Abstract

The aim of this paper is to describe a case report of EMP in an HIV-positive patient. A 44-year-old, dark-skinned HIV-infected woman was referred to the Oral Diseases Treatment Center with a swelling at palate and left gingival fornix in the maxilla. Biopsy was taken and the oral lesion was diagnosed as EMP with well-differentiated plasma cells and restriction of the lambda light-chain. Skeletal survey was performed and no radiograph alterations were observed, thus supporting the diagnosis of EMP. Patient was referred to treatment and after two months of chemo and radiotherapy, an expanding lesion was observed in L5/S1 patient's vertebrae. Biopsy of the spinal lesion was consistent with lymphoma with plasmocitary differentiation, supporting the diagnosis of multiple myeloma (MM). Regarding the medical history, the final diagnostic was an oral extramedullary plasmacytoma with rapid progression into multiple myeloma. It is crucial to emphasize the relevance of HIV infection as a risk factor for both aggressive clinical behavior and unusual clinical presentation of extramedullary plasmacytoma cases.

## 1. Introduction

According to the World Health Organization (WHO), extramedullary plasmacytoma (EMP) is a monoclonal plasmatic soft-tissue proliferation, without bone marrow involvement. It is a tumor composed almost exclusively of plasma cells arranged in clusters or sheets with a scant, delicate, supportive, and connective tissue stroma [1, 2].

Extramedullary plasma cell tumors occur in a wide variety of organs and tissues. However, it has been reported in head and neck of more than 80% of the cases, usually in the nasal cavity with associated bone destruction [3, 4]. Extramedullary plasmacytomas vary considerably in size, the diameter ranging from one to several centimeters. They are usually well limited, firm, and spherical, but they may be lobulated, pedunculated, or polypoid and show evidence of infiltration. The great majority are yellow-gray with a red cut surface, while some of the other tumors have a blue-red appearance. Involved regional lymph nodes are firm, gray white, and may measure up to 3 cm. The symptoms are those due to pressure and obstruction [5]. The tumor is usually highly sensitive to radiotherapy, and most cases do not progress into multiple myeloma [3, 6]. Recently, Ngolet et al. [7] reported that a secondary metastatic cutaneous plasmacytoma is a multiple extramedullary plasma cell proliferation involving skin. Its occurrence was associated with advanced myeloma and a poor prognosis.

Over the last 10 years, it has become apparent that the spectrum of malignant diseases associated with human immunodeficiency virus (HIV) has been expanding [8]. Plasma cell tumors are extremely rare in this group of patients [9] and it has been found that these patients are younger and they present a greater tendency to develop solitary extramedullary plasmacytoma with atypical clinical evolution and greater aggressiveness of the neoplastic process [10]. It has a shorter latency period and often has extramedullary involvement with unusual clinical presentation [11–13]. There are only few cases of extramedullary plasmacytoma of the head and neck region associated with HIV-positive patients published in the literature. Therefore, the aim of this paper is to present case report of an HIV-positive patient diagnosed for extramedullary plasmacytoma.

## 2. Case Report

A 44-year-old, dark-skinned woman was referred to the Oral Disease Treatment Center of São Leopoldo Mandic Dental School, Campinas/Brazil, with a complaint of difficulty in wearing her dentures. Her medical history revealed HIV infection, with irregular use of antiretroviral therapy. Patient also reported multiple sexual partners and use of injection drugs, cocaine, crack, and marijuana.

**Figure 1:**
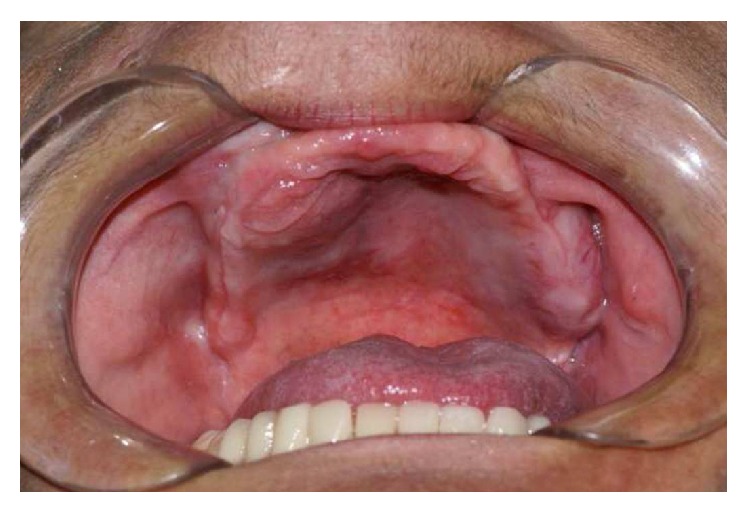
Clinical intraoral presentation at time of the first appointment.

Clinical examination revealed an asymptomatic swelling at right gingival sulcus in the maxilla (Figure 1).

**Figure 2:**
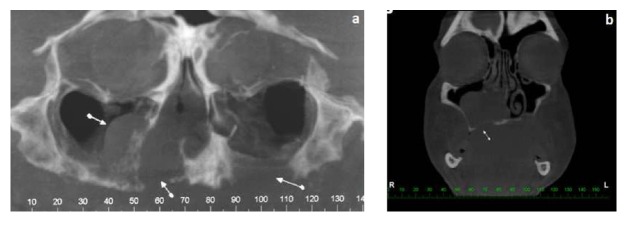
Extent of bone destruction seen on CT scans ((a) CT, axial view; (b) CT, coronal view).

Computed tomography scan revealed a solid tumor mass on the floor of the nasal cavity, measuring 5.6 × 5.2 × 5.2 cm, leading to erosion of the hard palate and of the medial wall of the maxillary sinus, bilaterally (Figure 2). No involvement of cervical lymph nodes was present.

**Figure 3:**
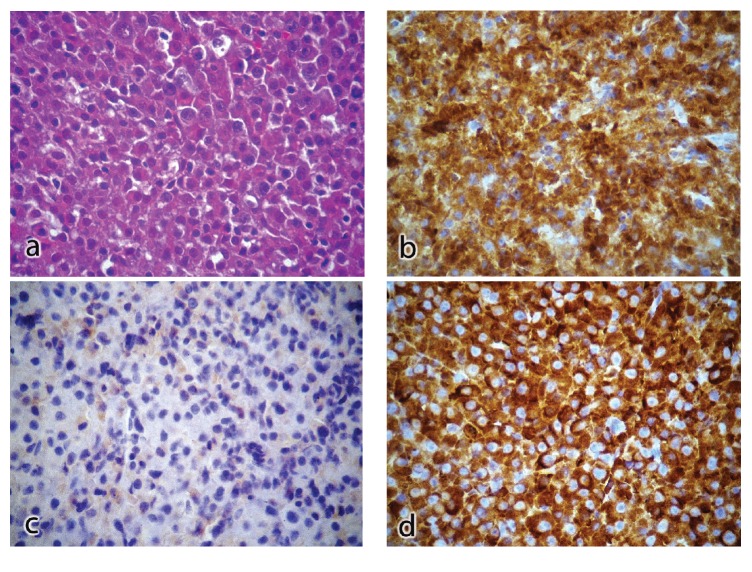
Microscopic examination revealed a plasma cell tumor. The histological sections showed lymphoid origin of tumor fragment characterized by the proliferation of atypical plasma cells which are arranged in sheets (a, HE); immunohistochemical reactions were positive for plasm cell (b); negative for kappa (c); and positive for lambda (d).

Diagnostic hypotheses were lymphoma, osteosarcoma, and malignant salivary gland neoplasia. Fine needle aspiration and incisional biopsy were performed at the same day of the initial appointment. Microscopic analysis revealed a neoplasm of well-differentiated plasma cells, with restriction of the lambda light-chain (Figure 3). Immunohistochemistry (IHC) showed positivity for CD138 and EMA in the neoplasm; CD79a in a strong and diffuse pattern through the neoplasm; CD56 in the membrane; and lambda in a focal pattern in the neoplasm. IHC was negative for kappa, IgG, and IgM. Bone marrow biopsy revealed reactional lymphoplasmacytic infiltrate and absence of infiltrative neoplastic cells. IHC showed positivity for CD20, CD3, kappa, and lambda (Figure 3).

In addition, total serum proteins, albumin-globulin ratio, and all other laboratory tests were within normal limits. No Bence Jones protein was found. Skeletal survey was performed, with no alterations on plain radiographs. These findings, in association with clinical data, led to the definitive diagnosis of EMP.

**Figure 4:**
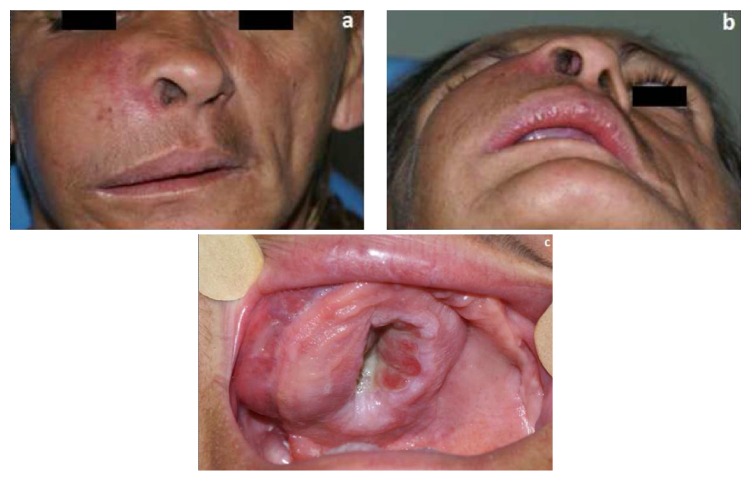
Clinical presentation at time of tumor staging ((a) fontal view; (b) coronal view; (c) intraoral view).

After the diagnosis procedure, the tumor was classified as T4N0M0 (Figure 4) and the patient was submitted to chemotherapy with thalidomide, dexamethasone, and pamidronate.

**Figure 5:**
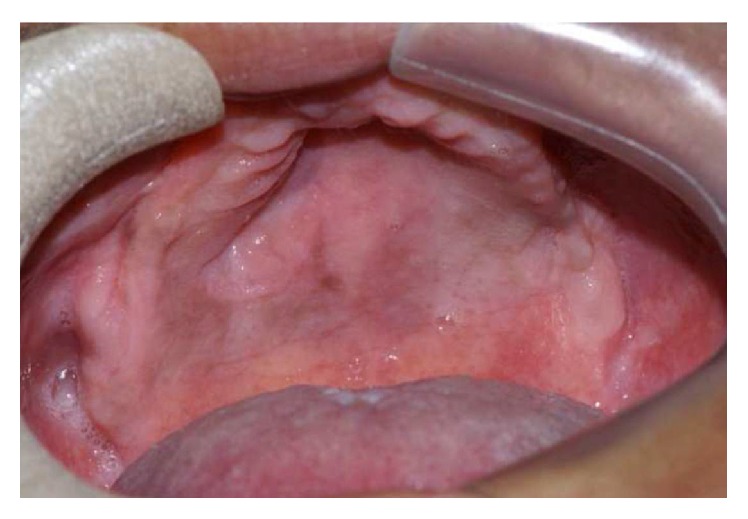
Clinical intraoral presentation after two months of chemo-radiotherapy treatment.

The tumor was also treated by radiation a total dosage of 42 Gy and a fraction size of 200 cGy during 2 months. When examined 3 months later, the nasal obstruction was relieved completely and no residual tumor was observed (Figure 5).

However, two months later, the patient developed sensitive and motor numbness in the lower limbs. Magnetic resonance imaging showed an expanding lesion in L5/S1 vertebrae, presenting medulla compression. Biopsy of the spinal lesion was performed, and microscopic features were consistent with lymphoma with plasmocitary differentiation. IHC was positive for CD20, CD3, and lambda and negative for kappa.

According to World Health Organization Classification of Tumors [1], the diagnosis of a plasmacytoma on biopsy and the presence of lytic bone lesions show multiple myeloma diagnosis (MM). The patient was submitted to chemotherapy with rituximab, cyclophosphamide, doxorubicin, vincristine, and prednisolone but discontinued treatment and quit attending the medical appointments. Information was later obtained that the patient had deceased for unknown causes, probably due to complications of the untreated multiple myeloma.

## 3. Discussion

The present study reports a case of EMP with atypical features which have been associated with the presence of HIV infection. Such neoplasms in this group of patients are extremely rare [9]. In addition, this tumor shows a different clinical behavior among HIV patients: occurrence in a younger age group, with a shorter latency period, with often extramedullary involvement and in a more aggressive clinical course, with a poor prognosis due to the poor immunity of the patient [9, 11–14]. Indeed, the mean of age of EMP is 60 years in noninfected patients [2], but in HIV-positive patients the mean age is 33 years [15]. In this study, the patient was 44 years-old.

Although a male predominance has been reported for EMP [5], in this study the patient was female. Other characteristics of the case herein reported are consistent with the ones described in the literature. Monoclonal gammopathies have a higher incidence among dark-skinned individuals, and EMP has been reported to occur more frequently in nasal cavity, nasopharyngeal, and paranasal sinus [1, 3, 16, 17]. In this study, the patient was dark skinned, and it is likely that the lesion was originated from the nasal cavity. However, the size of the lesion (bigger than 5 cm), at the time of diagnosis, makes it difficult to define a precise location.

According to Joseph et al. [10], differentiating between plasmablastic lymphoma, plasma cell myeloma, and the solitary EMPs is in itself a diagnostic challenge. EMP has been treated with surgical excision, radiotherapy, chemotherapy, or combined surgery and radiotherapy [9]. EMP is a highly radiosensitive lesion; however, no firmly dose-response relationship has been established due to small patient series and low local failure rates [6]. An optimal radiation dosage appears to be in the range of 40–50 Gy [18]. In the present case, a complete regression of the EMP was obtained following a total dosage of 42 Gy of radiotherapy.

The case presented in this study showed a rapid evolution into MM, only 6 months after the initial diagnosis, in an aggressive clinical behavior. It is estimated that 20% to 36% of the cases of EMP can progress into MM [1, 3, 4, 17]. However, in HIV-positive patients, plasma cell tumors may present at unusual sites and progress rapidly to involve multiple sites, including the soft tissues and viscera [19].

Previous studies have investigated the risk factors that would predispose patients with solitary plasmacytoma to disease progression. Thus, in age below 60 years, extramedullary localization and radiotherapy have been related to a 10-year disease-free survival [4]. On the other hand, unfavorable factors for MM development were identified on people older than 60 years and bone localization [4].

The case reported here presented all factors for a favorable outcome and even so showed an aggressive evolution of EMP into MM and eventually death. Such outcome emphasizes the relevance of HIV infection in a more aggressive clinical cause and, eventually, a poor prognosis in patients with EMP [8, 11, 19].

The proposed mechanism for this clinical behavior in HIV-positive patients is related to impaired T-cell function, deregulation, and hyperactivity of B cells. These factors, associated with persistent antigenic stimulation, could encourage transformation of stimulated B cells into malignant plasma cells [15]. However, the final outcome of the case herein reported cannot be adequately discussed since the patient discontinued her treatment after the diagnosis of MM. So far there have been no large studies reporting an optimal therapy for myeloma and other plasma cell dyscrasias in the HIV-positive population [14].

Concerning the microscopic features, the WHO diagnostic criteria state that EMP shows identical microscopic and immunophenotype features as those of plasma cell myeloma [1]. Microscopic features frequently show plasma cells morphology, and IHC may show expression of EMA, an epithelial membrane antigen of plasma cells; of CD56/58, a natural killer antigen; of the immunoglobulin-associated antigen CD79a; and of CD138, a reliable marker for identifying and quantifying normal and tumoral plasma cells in paraffin sections [1, 19]. Light-chain immunoglobulins are identified in 11% of the cases of plasma cell tumors, with a higher prevalence of the kappa light-chain [20]. In the case reported here, unusual microscopic features were observed, with a predominance of lambda light-chain among monoclonal gammopathies.

Regarding previous studies there is emphasis on the association of HIV infection, EMP, and MM. Ngolet et al. [7] described a secondary metastatic cutaneous plasmacytoma as a multiple extramedullary plasma cell proliferation involving skin. Its occurrence was associated with advanced myeloma. According to Hazarika et al. [21], in view of high incidence of progression to MM in due course the patients should be kept under constant surveillance. However, further studies are required to identify risk factors that correlate EMP and its rapid progression into MM.

## 4. Conclusion

In conclusion, this study reports a case of EMP in an HIV-positive patient. It is important to observe the association of HIV infection and a higher incidence of these lesions, as well as its aggressive clinical behavior and unusual clinical presentation.
